# Giant sialolith after submandibulectomy

**DOI:** 10.11604/pamj.2016.25.228.10717

**Published:** 2016-12-07

**Authors:** Aryé Weinberg, Andreas Eberhard Albers

**Affiliations:** 1 Prosper-Hospital, Department of Otorhinolaryngology, Head and Neck Surgery, Recklinghausen, Germany; 2 Charité Universitätsmedizin Berlin, Department of Otorhinolaryngology, Head and Neck Surgery, Campus Benjamin Franklin, Berlin, Germany

**Keywords:** Giant sialolith, submandibulectomy, wharton duct

## Image in medicine

A 59-year-old Patient presented with cerivical lymphnode and a submandibular swelling on the right side of unknown cause that was not responsive to antibiotic treatment. 16 years ago a submandibulectomy was performed on the right side, nevertheless submandibular swellings had occurred repeatedly. Clinical examination showed a right-sided cervical swelling in the submandibular region. The floor of the mouth was indurated and pus could be squeezed out of the wharton’s duct. A computed tomography of the neck revealed a calcified structure of 3.8 x 1.3 cm located anterior of the former submandibular region (A). Therapeutically the residual Wharton duct containing the sialolith was removed (B). Giant sialoliths after submandibulectomy are extremely rare. If parts of the Wharton duct remain, sialolithiasis may occur spontaneously or become evident after growth due to further calcification. The treatment of choice consists of the excision of the residual duct with the sialolith and any remaining gland tissue.

**Figure 1:**
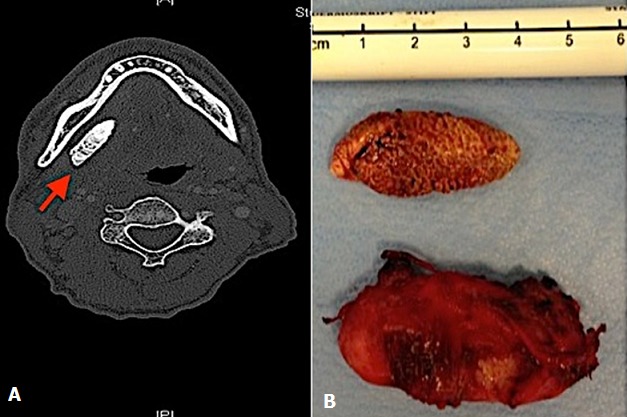
A) ct-scan showing the giant sialolith; B) wharton duct and giant sialolith

